# Circulating biomarkers of immunity and inflammation, risk of Alzheimer’s disease, and hippocampal volume: a Mendelian randomization study

**DOI:** 10.1038/s41398-021-01400-z

**Published:** 2021-05-17

**Authors:** Lana Fani, Marios K. Georgakis, M. Arfan Ikram, M. Kamran Ikram, Rainer Malik, Martin Dichgans

**Affiliations:** 1 Department of Epidemiology, Erasmus MC University Medical Center, Rotterdam, The Netherlands; 2 Institute for Stroke and Dementia Research, University Hospital LMU Munich, Munich, Germany; 3 Department of Neurology, Erasmus MC University Medical Center, Rotterdam, The Netherlands

**Keywords:** Biomarkers, Psychiatric disorders

## Abstract

The aim of this study was to explore the association between genetically predicted circulating levels of immunity and inflammation, and the risk of Alzheimer’s disease (AD) and hippocampal volume, by conducting a two-sample Mendelian Randomization Study. We identified 12 markers of immune cells and derived ratios (platelet count, eosinophil count, neutrophil count, basophil count, monocyte count, lymphocyte count, platelet-to-lymphocyte ratio, monocyte-to-lymphocyte ratio, CD4 count, CD8 count, CD4-to-CD8 ratio, and CD56) and 5 signaling molecules (IL-6, fibrinogen, CRP, and Lp-PLA2 activity and mass) as primary exposures of interest. Other genetically available immune biomarkers with a weaker a priori link to AD were considered secondary exposures. Associations with AD were evaluated in The International Genomics of Alzheimer’s Project (IGAP) GWAS dataset (21,982 cases; 41,944 controls of European ancestry). For hippocampal volume, we extracted data from a GWAS meta-analysis on 33,536 participants of European ancestry. None of the primary or secondary exposures showed statistically significant associations with AD or with hippocampal volume following *P-*value correction for multiple comparisons using false discovery rate < 5% (*Q*-value < 0.05). CD4 count showed the strongest suggestive association with AD (odds ratio 1.32, *P* < 0.01, *Q* > 0.05). There was evidence for heterogeneity in the MR inverse variance-weighted meta-analyses as measured by Cochran *Q*, and weighted median and weighted mode for multiple exposures. Further cluster analyses did not reveal clusters of variants that could influence the risk factor in distinct ways. This study suggests that genetically predicted circulating biomarkers of immunity and inflammation are not associated with AD risk or hippocampal volume. Future studies should assess competing risk, explore in more depth the role of adaptive immunity in AD, in particular T cells and the CD4 subtype, and confirm these findings in other ethnicities.

## Introduction

The immune system is increasingly recognized to be involved in the pathogenesis of Alzheimer’s disease (AD)^1,2^. Recent genome-wide association studies (GWASs) have established AD risk loci within or near genes that are expressed in microglia^3^. This led to the concept of the innate immune system being involved in the early steps of the disease and, thus, much effort has been dedicated in studying innate immunity in relation to AD. A recent meta-analysis of observational studies revealed that the immune-related signaling molecules C-reactive protein (CRP), interleukin (IL)-6, α1-antichymotrypsin, lipoprotein-associated phospholipase A2 (Lp-PLA2) activity, and fibrinogen were each associated with risk of all-cause dementia^4^. Less is known about the contribution of the adaptive immune system in relation to AD, but a recent observational study discovered clonally expanded CD8^+^ T-effector memory cells in the cerebrospinal fluid of AD patients, revealing an adaptive immune response in AD^5^. Moreover, we previously found that higher levels of innate immune cells lead to a higher dementia risk, whereas higher levels of adaptive immune cells are protective for developing dementia^6^. Given the observational design of the majority of available studies and the difficulty of studying the effect of the immune system on AD in a trial, it is uncertain whether the observed associations are causal, i.e., independent of other risk factors, and not biased by reverse causation^7^.

Mendelian randomization (MR) exploits genetic variants influencing the exposure of interest as unbiased proxies for the exposure, i.e., instruments, to infer causality^8^. To our knowledge, there are only few MR studies performed where the association between circulating biomarkers of immunity and inflammation, and dementia, was studied^9–13^. Moreover, a large GWAS meta-analysis on hippocampal volume^14^ allows exploration of these biomarkers with hippocampal volume as imaging endophenotype of AD, which, to date, has not been performed. Furthermore, as GWAS studies are increasing in size, the number of instruments that can be used to estimate the causal effect of a risk factor on an outcome also increases. This could lead to more heterogeneity among the causal estimates obtained from multiple genetic variants, pointing to a possible violation of the necessary instrumental variable assumptions, but also to a scenario in which causal estimates based on each variant in turn differ more strongly than expected by chance alone. These variants could then be divided into distinct clusters, such that all variants in the cluster have similar causal estimates. There are now novel techniques available, which allow for cluster analyses of variants, which can capture distinct causal mechanisms by which a risk factor influences an outcome with different magnitudes of causal effect^15^.

Here, by leveraging data from large-scale genomic studies on circulating biomarkers of immunity and inflammation, and the large AD dataset from The International Genomics of Alzheimer’s Project (IGAP) GWAS^3^ and hippocampal volume GWAS^14^, we implemented a two-sample MR study to (1) explore the associations between genetic predisposition to higher or lower circulating levels of immune cells^12,16^ and signaling molecules^13,17–19^ with risk of AD; (2) explore the associations between genetic predisposition to these biomarkers with hippocampal volume; (3) explore the associations between genetic predisposition to circulating biomarkers of immunity and inflammation with limited a priori evidence with AD and hippocampal volume; and (4) explore whether different genetic variants influence the exposures, and thus AD and hippocampal volume in distinct ways by performing cluster analyses.

## Methods

### Study design, data sources, and genetic instrument selection

Table 1 summarizes the data sources used in the current MR study. The genetic instruments were taken from publicly available summary statistics. For each of the circulating immunity traits, we selected single-nucleotide polymorphisms (SNPs) associated with their circulating levels at a genome-wide threshold of significance (*P* < 5 × 10^−8^). After extracting the summary statistics for significant SNPs, we pruned all SNPs in linkage disequilibrium (LD) (*r*^2^ < 0.01 in the European 1000 Genomes Project reference panel), retaining SNPs with the lowest *P*-value as an independent instrument. For some exposures (IL-6, CRP, Lp-PLA2, 23 cytokines, and IL-1), we used previously selected instruments (Table 1). We identified independent SNPs significantly associated with circulating biomarker levels of immunity and inflammation, and merged these with the outcome datasets; the SNPs that were also available in the outcome datasets were used as final instruments for analysis. As all analyses are based on publicly available summary statistics and not individual-level data, no ethical approval from an institutional review board was required.

**Table 1:** Study design and data sources MR.

Primary exposures (biomarkers of immunity and inflammation with strong a priori evidence)					Instruments
Discovery GWAS	Phenotype	Sample size	Ancestry	Adjustments	
**Immune cells**					
UK Biobank/UK BiLEVE/INTERVAL	Lymphocyte count, granulocyte count, platelet count, monocyte count, basophil count, eosinophil count	171,643 Individuals	European	Age, sex, body mass index, alcohol consumption, and smoking status	As performed by Astle et al.^12^
NTR	Neutrophil-to-lymphocyte ratio (NLR), platelet-to-lymphocyte ratio (PLR)	5901 Individuals	European	Age, sex, and genotype platform	Only instruments for PLR were available^16^
NTR	Monocyte-to-lymphocyte ratio	5892 Individuals	European	Age, sex, principal components, genotype platform	As described by Lin et al.^45^
International HIV Controllers Study	CD4 : CD8 lymphocyte ratio, CD3, CD4, CD8-positive T and CD19-positive B lymphocytes	2538 Individuals	European	Age and sex effects	Derived from summary statistics from Ferreira et al.^46^
**Signaling molecules**					
INTERVAL Study/CHARGE Inflammation Working Group	IL-6	204,402 Individuals	European	Age, sex, duration between blood draw and processing, population structure	As selected by Georgakis et al.^17^
CHARGE Inflammation Working Group^18^	Fibrinogen levels	120,246 Individuals	European	Age, sex, population structure	As selected by Ward-Caviness et al.^47^
CHARGE Inflammation, Working Group	CRP levels	204,402 Individuals	European	Age, sex, population structure	As selected by Georgakis et al.^17^
CARDIoGRAM Consortium^48^	Lp-PLA2 activity/mass	12,126 Individuals	European	Age, sex	As selected by Casas et al.^19^

Secondary exposures (biomarkers of immunity and inflammation with limited a priori evidence)					Instruments
Discovery GWAS	Phenotype	Sample size	Ancestry	Adjustments	
FINRISK/CRYFS	Circulating levels of 41 cytokines and growth factors	8293 Iindividuals	Finnish	Age, sex, BMI	For 23 cytokines as selected by Georgakis et al.^22^
Cardiovascular Health Study (>65 years)/InCHIANTI^49^	IL-1	4500 Individuals	European	Age, gender, and the first 10 principal components reflecting background ancestry	As selected by Freitag et al.^50^
CHARGE^51^	ICAM-1	9813 Individuals	European	Age and sex, sampling design	From genome-wide significant SNPs
Women’s Genome Health Study	ICAM-1	22,435 Women	European	Sub-Caucasian ancestry, age, menopause, smoking, BMI	Used only the 4 novel loci that have been replicated in CHARGE^52^
CHARGE^51^	P-selectin	4115 Genome-wide significant SNPs	European	Age and sex, sampling design	From genome-wide significant SNPs

Primary outcomes					Source
Discovery GWAS	Phenotype	Sample size	Ancestry	Adjustments	
IGAP	Alzheimer’s disease	21,982 Cases, 41,944 controls	European	Sex and age	^53^
ENIGMA and the CHARGE consortia	Hippocampal volume	33,536 Individuals	European	4 MDS components, age^2^, sex, intracranial volume and diagnosis (when applicable)	^14^

GWAS names: *CARDIoGRAM* Coronary ARtery DIsease Genome-wide Replication and Meta-analysis, *CHARGE* Cohorts for Heart and Aging Research in Genomic Epidemiology, *CRYFS* Cardiovascular Risk in Young Finns Study, *InCHIANTI* aging in the Chianti area, *IGAP* International genomics of Alzheimer’s project, *NTR* Netherlands Twin Register, *UK BiLEVE* UK Biobank Lung Exome Variant Evaluation.

Phenotypes: *CD* cluster of differentiation, *CRP* C-reactive protein, *ICAM-1* intercellular adhesion molecule 1, *IL* interleukin, *Lp-PLA2* lipoprotein-associated phospholipase A2.

### Primary exposures (biomarkers of immunity and inflammation with strong a priori evidence)

To minimize weak instrument bias and maximize power, we carefully selected our primary exposures prior to data analysis based on the underlying GWAS size, population characteristics, and a priori evidence for the associations with AD (Table 1)^20^. We identified 12 immune cells and derived ratios (platelet count, eosinophil count, neutrophil count, basophil count, monocyte count, lymphocyte count, platelet-to-lymphocyte (PLR) ratio, monocyte-to-lymphocyte ratio, CD4 count, CD8 count, CD4 : CD8 ratio, and CD56) and 5 signaling molecules (IL-6, fibrinogen, CRP, and Lp-PLA2 activity and Lp-PLA2 mass) as primary exposures of interest.

### Secondary exposures (biomarkers of immunity and inflammation with limited a priori evidence)

Other immune-related exposures, for which there are less validated biomarkers of immunity and inflammation or less valid instruments available, were selected as secondary exposures (Table 1). More specifically, a GWAS identified multiple common genetic variants that influence circulating levels of 41 cytokines and growth factors^21^, of which we used pre-selected instruments for 23 cytokines or growth factors that were not in LD and not associated with levels of >1 cytokine^22^. These instruments have a weaker a priori link to AD and were therefore selected as secondary exposures. Furthermore, we used genetic instruments for IL-1, intercellular adhesion molecule 1 (ICAM-1), and P-selectin as additional secondary exposures due to smaller powered underlying GWASs.

### Outcomes

The primary outcome for this study was AD defined by clinical diagnosis of AD. In addition, we looked at hippocampal volume as an imaging AD endophenotype as hippocampal degeneration is one of the pathological hallmarks of AD. We extracted estimates for the associations of the selected instruments with AD from IGAP GWAS dataset (21,982 cases; 41,944 controls of European ancestry)^3^. For hippocampal volume, we extracted data from publicly available summary statistics of the Cohorts for Heart and Aging Research in Genomic Epidemiology–Enhancing Neuro Imaging Genetics through Meta Analysis GWAS meta-analysis on 33,536 participants of European ancestry^14^.

Our power calculations^23^ revealed that based on the sample size of IGAP, we had sufficient power for most biomarkers of immunity and inflammation to detect meaningful effect sizes. Specifically, we had >80% power to detect significant associations with AD for 8 out of 12 immune cells and for 3 out of 5 signaling molecules at an effect size (odds ratio [OR]) of 1.10 or 0.90 (Supplementary Table S1). All markers were analyzed, even when potentially underpowered, to guide future research.

### Statistical analysis

We first extracted data and harmonized the effect alleles across GWASs. The MR association between each immune cell or signaling molecule and AD or hippocampal volume was then estimated using the Wald estimator and the delta method after pooling individual SNP MR estimates using inverse variance-weighted (IVW) meta-analysis^24^. Fixed-effect IVW was used in the absence of heterogeneity and random effects in the presence of heterogeneity (Cochran *Q*-derived *P* < 0.05). Statistical significance for the MR associations with AD and hippocampal volume were set at a *P-*value corrected for multiple comparisons using false discovery rate (FDR) < 5%. A *P* < 0.05, but above the FDR-corrected threshold, was considered as suggestive for an association. These analyses were repeated for the secondary exposures with AD and hippocampal volume, and we set a separate corrected *P-*value for multiple comparisons of secondary exposures using FDR < 5%.

Cochran’s *Q*-derived *P* < 0.05 from the IVW MR was used as indicator of possible horizontal pleiotropy^25^. For markers with >2 SNPs showing either significant or suggestive associations or significant heterogeneity in the primary IVW MR analysis, we conducted additional sensitivity analyses that vary in their underlying assumptions regarding the presence of pleiotropic genetic variants that may be associated with the outcome independently of the exposure. In particular, we used the weighted median method, which requires that at least half of the information for the MR analysis comes from valid instruments^26^. We also used the weighted mode approach, which requires that the largest number of similar (identical in infinite samples) individual-instrument causal effect estimates comes from valid instruments, even if the majority of instruments are invalid^27^. For consistency with other literature and to further relax the IVW assumptions, we used MR-Egger regression, which provides a consistent estimate of the causal effect, under a weaker assumption—the InSIDE (INstrument Strength Independent of Direct Effect) assumption^28^. In addition, we used the contamination mixture method, which is implemented by constructing a likelihood function based on the variant-specific causal estimates. If a genetic variant is a valid instrument, then its causal estimate will be normally distributed about the true value of the causal effect, but if a genetic variant is not a valid instrument then its causal estimate will be normally distributed about some other value^29^. We also tested for outlier SNPs using MR-Pleiotropy Residual Sum and Outlier^30^.

Finally, as it is possible that different genetic variants influence the risk factor in distinct ways, e.g., via distinct biological mechanisms, we further examined heterogeneity by performing cluster analyses using the MR-Clust package^15^. As recommended, we implemented this method conservatively, i.e., only assigning a variant to a cluster if the conditional probability of cluster assignment is ≥0.8 and only reporting a cluster if at least 4 variants satisfy this criterion. Variants that do not satisfy these criteria and that do not fit into a null cluster will be assigned to a “junk” cluster. Immune cells or signaling molecules that showed suggestive associations and for which more genome-wide significant SNPs were available were also explored for potential clustering of variants.

Statistical analyses were conducted in RStudio (R version 3.6.3).

## Results

### Primary exposures with AD

**Fig. 1:**
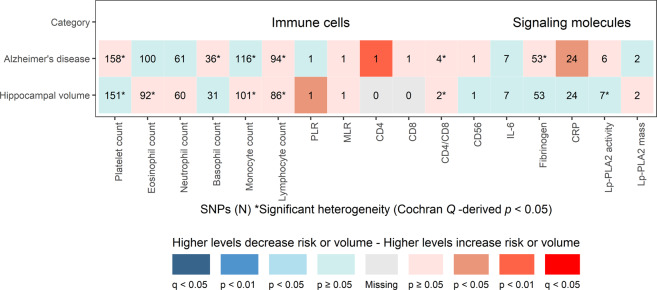
Primary Mendelian randomization associations of circulating immune cell and signaling molecule levels with Alzheimer’s disease and hippocampal volume. Shown are the results derived from the primary inverse variance-weighted meta-analysis. None of the immune cells or signaling molecules survived the multiple testing threshold of false discovery rate < 5% (*q* < 0.05). CD, cluster of differentiation; CRP, C-reactive protein; IL, interleukin; Lp-PLA2, Lipoprotein-associated phospholipase A2; MLR, monocyte-to-lymphocyte ratio; PLR, platelet-to-lymphocyte ratio.

The primary results of the MR analyses for the genetic variants of immune cells and signaling molecules with AD are presented in Fig. 1. Following *P-*value correction for multiple comparisons using FDR < 5% (*Q*-value < 0.05), none of the immune cells or signaling molecules showed statistically significant associations with AD. CD4 count showed the strongest suggestive association with AD by an OR of 1.32, *P* = 0.005, *Q* = 0.170 (*P* < 0.01, *Q* > 0.05) with the next strongest suggestive association being between CRP and AD with *P* = 0.029 (*P* < 0.05, *Q* > 0.05).

### Primary exposures with hippocampal volume

The primary results of the MR analyses for the immune cells and signaling molecules with hippocampal volume are presented in Fig. 1 and Supplementary Table S1. None of the immune cells or signaling molecules showed statistically significant associations with hippocampal volume following *P*-value correction for multiple comparisons. Only PLR ratio showed a suggestive *P* = 0.037 (*P* < 0.05, *Q* > 0.05) association with hippocampal volume.

### Secondary exposures with AD and hippocampal volume

**Fig. 2:**
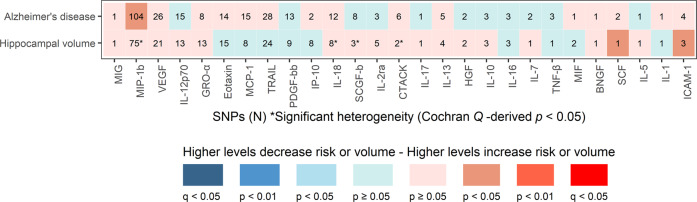
Secondary Mendelian randomization associations of circulating cytokines and growth factors with Alzheimer’s disease and hippocampal volume. Shown are the results derived from the secondary inverse variance-weighted meta-analysis. None of the immune traits survived the multiple testing threshold of false discovery rate < 5% (*q* < 0.05). BNGF, β-nerve growth factor; CTACK, cutaneous T-cell-attracting chemokine; GRO-α, growth-regulated oncogene α; HGF, hepatocyte growth factor; ICAM-1, intercellular adhesion molecule 1; IL, interleukin; IP-10, interferon γ-induced protein 10; MCP-1, monocyte chemoattractant protein-1; MIF, macrophage migration inhibitory factor; MIG, monokine induced by γ-interferon indicates; MIP-1b, macrophage inflammatory protein-1β; PDGF-bb, platelet-derived growth factor-bb; SCF, stem cell factor; SCGF-b, stem cell growth factor β; TRAIL, TNF-related apoptosis-inducing ligand; VEGF, vascular endothelial growth factor.

The secondary results of the MR analyses are presented in Fig. 2 and Supplementary Table S2. Similarly, none of the biomarkers of immunity and inflammation showed statistically significant associations with AD or hippocampal volume following *P*-value correction for multiple comparisons. MIP-1b showed a suggestive association with AD, with *P* = 0.024 (*P*-value < 0.05, *Q* > 0.05), whereas stem cell factor (*P* = 0.031) and ICAM-1 (*P* = 0.016) showed suggestive associations with hippocampal volume (*P* < 0.05 level, *Q* > 0.05).

### Sensitivity analyses

**Fig. 3:**
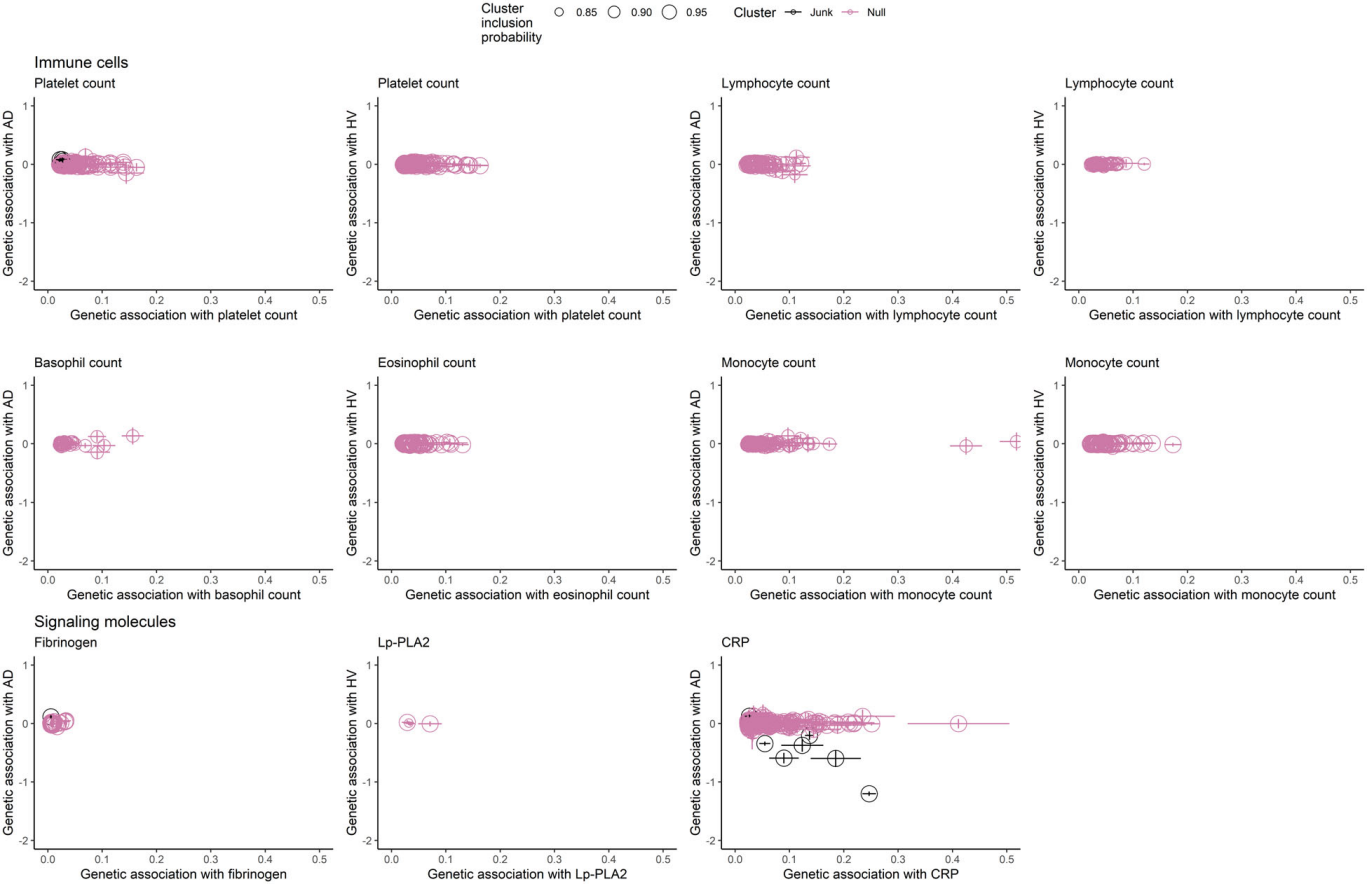
Exploration of heterogeneity by cluster analyses. Shown are the genetic associations for the individual variants with the exposure and outcome; lines indicate confidence intervals. When restricting to a cluster probability assignment of ≥0.8 and ≥4 variants per cluster, no clusters of variants were identified. AD, Alzheimer’s disease; CRP, C-reactive protein; HV, hippocampal volume; Lp-PLA2, lipoprotein-associated phospholipase A2. The junk cluster denotes variants with estimates that do not fit in any cluster.

There was evidence for heterogeneity in the primary and the secondary MR IVW analyses as measured by Cochran *Q*. Alternative tests furthermore revealed varying estimates changing direction for multiple exposures (Supplementary Table S3). Cluster analyses did not reveal clusters of variants (Fig. 3).

## Discussion

Exploring genetically predicted circulating biomarkers of immunity and inflammation in an adequately powered two-sample MR approach involving the largest GWAS datasets available, we found no association between genetically predicted circulating levels of immune cells and signaling molecules as primary exposures and AD or hippocampal volume. Similarly, none of the secondary exposures including genetically predicted levels of biomarkers of immunity and inflammation showed statistically significant associations with AD or hippocampal volume. Sensitivity analyses showed evidence for heterogeneity, but we found no clustering of variants.

Our findings are in contrast with observational studies that reported on significant associations between several circulating blood biomarkers of signaling molecules, immune cells, and AD^4,6^. For example, we expected to find an association between higher platelet count and higher AD risk, as we previously found that an increase of circulating platelets as measured in the blood over time increased AD risk^6^. However, if the risk factor is a protein biomarker, such as CRP, we can select genetic variants located in or around the coding region for that protein as instruments. This is more difficult for polygenic risk factors such as platelets, as the influence of genetic variants on such a risk factor is unlikely to be specific^31^. Indeed, we found substantial heterogeneity when studying immune cells and signaling molecules, but could not find meaningful clusters of genetic variants that could have a distinct effect on the risk factor, supporting the conclusion that our findings are truly null. On the other hand, our power calculations revealed that some analyses were underpowered to detect significant associations, e.g., for platelet count and AD. However, these exposures in particular did not even show suggestive associations with AD, implying that estimates are very small and probably—even if sufficiently powered—would not have survived correction for multiple testing.

The strongest suggestive association we found in our study was between CD4 cell count and AD, where higher levels of CD4 cell count increase AD risk, although only one SNP could be used as an instrument. This suggestive association is unexpected, as HIV-associated dementia is accompanied by a lower CD4 cell count^32^. However, it is recognized that T lymphocytes play a central role in the pathogenesis of multiple sclerosis (MS), with CD4+ T cells predominating in acute MS lesions^33^. Combined with the recent finding that clonally expanded CD8^+^ T-effector memory cells have been found in the cerebrospinal fluid of AD patients^5^, the role of adaptive immunity in AD, in particular T cells and the CD4 subtype, is worth investigating further in relation to AD.

In contrast to our findings, one MR study^13^ found a protective effect of CRP on AD. An explanation for this difference could be the selection of instruments for CRP. In our study, we used 24 SNPs as instruments that are gene specific for CRP, thereby reducing pleiotropy. When examining CRP further by performing a cluster analysis including all genome-wide significant SNPs, we found no clusters of variants, in particular no cluster forming a biologically meaningful protective pathway of CRP on AD.

Our study has limitations. First, we could not assess competing risk by, e.g., mortality in this study, which could generate paradoxical MR estimates^34^. Second, we cannot exclude that the additional adjustments for body mass index, alcohol consumption, and smoking status performed in the blood cell trait GWAS^12^ led to collider bias (i.e., a collider between a genetic variant and confounders of the risk factor-outcome association) during instrument selection. However, the potential impact of such collider bias is likely to be less than other biases^34^. Third, as we used multiple proposed instruments where effect heterogeneity is likely, effect estimates need to be interpreted with caution. Fourth, for some exposures, especially those reflecting adaptive immunity, we were limited by the few known genome-wide significant genetic variants influencing these traits, potentially leading to weak instrument bias. Targeted studies incorporating further GWAS data on individual circulating adaptive immune biomarkers might reveal additional associations not captured by our approach. Furthermore, despite using the largest available datasets, some of our analyses could be limited by power to detect small but functionally relevant causal effects. This lack of power applies to both the discovery of the exposure and to the outcome. Fifth, the IGAP GWAS dataset contains mainly clinically diagnosed cases of AD (only 8% of cases and controls are pathologically confirmed), thus potentially leading to misclassification of the outcome^35^. Similarly, although hippocampal atrophy is a hallmark feature of AD, a recently recognized disease entity named limbic-predominant age-related TDP-43 encephalopathy has shown to be mimicking Alzheimer’s type dementia, causing hippocampal and medial temporal lobe atrophy in more than 20% of old demented people^36^. Sixth, the underlying study populations were of European ancestry, limiting generalizability to other ethnicities. Finally, although we have tried to deal with these factors in our study, LD, pleiotropy, canalization, and population stratification remain potential flaws in the MR approach^37^.

### Future perspectives

SNP and biomarker studies investigating age-related diseases play a crucial role in unraveling the mechanisms underlying disease development. Apart from AD, examples of such studies include suggesting new targets for age-related macular degeneration, amyotrophic lateral sclerosis, and other neurodegenerative disorders^38^. These various targets need validation in suitable animal models. Creating these animal models can be challenging, as it requires undertaking standardization^39^. The blood–brain barrier forms an additional layer of complexity, as drug molecules are unable to reach the brain^40^. Nevertheless, the SNP animal model therapeutics field provides an excellent framework for studying interventions reducing risks. The pace of translation in the field of AD could be accelerated by understanding the causative events and mechanisms in the pathogenesis of AD using this framework. Integrating MR analysis when undertaking such studies could aid in the clinical translation, combined with other techniques involving genetics^41–43^. Despite the many successes in the field of genetics, in total only 53% of phenotypic variance is explained, with known AD SNPs only explaining 31% of the genetic variance^44^. Thus, the whole spectrum of research, including non-genetics, is needed in order to detect the functional ways to underpin the association between the immune system and the physiopathologic network that facilitates the manifestation of AD. In conclusion, this study suggests that genetically predicted circulating biomarkers of immunity and inflammation are not associated with AD risk or hippocampal volume. Future studies should assess competing risk, explore in more depth the role of adaptive immunity in AD, in particular T cells and the CD4 subtype, and confirm these findings in other ethnicities.

## Supplementary information

Circulating biomarkers of immunity and inflammation and risk of Alzheimer’s disease and hippocampal volume: A mendelian randomization study
