# Supplementary material for: Circulating biomarkers of immunity and inflammation, risk of Alzheimer’s disease, and hippocampal volume: a Mendelian randomization study
Source: Transl Psychiatry. 2021 May 17;11:291. doi: 10.1038/s41398-021-01400-z (PMC8129147; doi:10.1038/s41398-021-01400-z)
Supplement: Supplementary file 1 — Circulating biomarkers of immunity and inflammation and risk of Alzheimer’s disease and hippocampal volume: A mendelian randomization study [file 41398_2021_1400_MOESM1_ESM.docx]

## Supplementary material

Circulating biomarkers of immunity and inflammation and risk of Alzheimer’s disease and hippocampal volume: A mendelian randomization study

Immunity and Alzheimer’s disease

Lana Fani MD^a^, Marios K. Georgakis MD PhD^b^, M. Arfan Ikram^a^ MD PhD, M. Kamran Ikram^a,c^ MD PhD, Rainer Malik PhD^b^, Martin Dichgans MD^b^

^a^Department of Epidemiology, Erasmus MC University Medical Center, Rotterdam, the Netherlands

^b^Institute for Stroke and Dementia Research, University Hospital LMU Munich, Munich, Germany ^c^Department of Neurology, Erasmus MC, the Netherlands

| **Exposure** | **SNPs (N)** | **Variance explained (R^2^)** | **Odds Ratio found in this study** | **Odds Ratio * for α <0.05 and [1-β] >0.8** |
| --- | --- | --- | --- | --- |
| *Immune cells* | | | | |
| Platelet count | 158 | 0.124 | 1.005 | 1.010 |
| Eosinophil count | 100 | 0.064 | 0.950 | 0.930 |
| Neutrophil count | 61 | 0.038 | 0.941 | 0.911 |
| Basophil count | 36 | 0.015 | 1.172 | 1.161 |
| Monocyte count | 116 | 0.100 | 0.967 | 0.944 |
| Lymphocyte count | 94 | 0.055 | 1.035 | 1.081 |
| PLR | 1 | 0.119 | 0.999 | 0.948 |
| MLR | 1 | 0.000 | 1.423 | 12.000 |
| CD4 count | 1 | 0.016 | 1.317 | 1.156 |
| CD8 count | 1 | 0.022 | 1.015 | 1.131 |
| CD4 to 8 ratio | 4 | 0.130 | 1.043 | 1.052 |
| CD56 count | 1 | 0.316 | 1.102 | 1.033 |
| *Signaling molecules* | | | | |
| IL-6 | 7 | 0.008 | 0.926 | 0.816 |
| Fibrinogen | 53 | 0.002 | 1.614 | 1.505 |
| CRP | 24 | 0.061 | 1.106 | 1.077 |
| Lp-PLA2 activity | 6 | 0.003 | 1.145 | 1.400 |
| Lp-PLA2 mass | 2 | 0.001 | 0.610 | 0.751 |
| *Shown are the Odds Ratios per 1 unit increase in circulating levels of the immune cells/signaling molecule, for which there is power (1-β) ≥80% to detect an existed association at a type I error of α <0.05. | | | | |
| 𝑅^2^ = (𝑏𝑒𝑡𝑎 𝑥 √2 𝑥 𝑀𝐴𝐹(1 − 𝑀𝐴𝐹))^2^, where MAF is the minimum allele frequency and beta is the effect of the SNP on the respective immune cell or molecule levels (Park et al 2010, Nat. Genet. 42, 570–575). Total variance was calculated in an additive model assuming no interaction between the individual SNPs. | | | | |

**Table S1. Characteristics of the genetic instruments selected for the circulating levels of immune cells and signaling molecules, variance explained by the selected instruments and power calculations for the Mendelian randomization study based on the sample sizes of the IGAP dataset.**

| **Table S2. Results of secondary IVW MR analyses of the associations between cytokines and growth factors with Alzheimer’s disease and hippocampal volume.** | | | | | | | | | |
| --- | --- | --- | --- | --- | --- | --- | --- | --- | --- |
| **Outcome** | **Exposure** | **IVW Estimate** | **95% CI** | | ***P*-value** | **Heterogeneity** | **Cochran *Q*-derived *P*** | **SNPs** | **FDR-corrected *P*** |
| Alzheimer's disease | MIG | 0.157 | -0.044 | 0.359 | 0.126 | - | - | 1 | 0.906 |
| Hippocampal volume | MIG | 0.087 | -0.035 | 0.210 | 0.161 | - | - | 1 | 0.906 |
| Alzheimer's disease | MIP-1b | 0.026 | 0.003 | 0.049 | 0.024 | 102.599 | 0.493 | 104 | 0.552 |
| Hippocampal volume | MIP-1b | 0.003 | -0.014 | 0.020 | 0.744 | 98.840 | 0.029 | 75 | 0.946 |
| Alzheimer's disease | VEGF | 0.017 | -0.022 | 0.055 | 0.405 | 19.211 | 0.787 | 26 | 0.906 |
| Hippocampal volume | VEGF | 0.003 | -0.021 | 0.027 | 0.805 | 20.223 | 0.444 | 21 | 0.953 |
| Alzheimer's disease | IL-12p70 | -0.025 | -0.078 | 0.028 | 0.360 | 9.125 | 0.823 | 15 | 0.906 |
| Hippocampal volume | IL-12p70 | 0.010 | -0.023 | 0.042 | 0.558 | 14.847 | 0.250 | 13 | 0.906 |
| Alzheimer's disease | GRO-α | 0.011 | -0.032 | 0.054 | 0.612 | 11.118 | 0.134 | 8 | 0.906 |
| Hippocampal volume | GRO-α | 0.012 | -0.014 | 0.038 | 0.383 | 8.691 | 0.276 | 13 | 0.906 |
| Alzheimer's disease | Eotaxin | 0.015 | -0.047 | 0.077 | 0.637 | 14.952 | 0.310 | 14 | 0.906 |
| Hippocampal volume | Eotaxin | -0.021 | -0.058 | 0.016 | 0.273 | 14.299 | 0.282 | 15 | 0.906 |
| Alzheimer's disease | MCP-1 | 0.009 | -0.052 | 0.070 | 0.771 | 21.518 | 0.089 | 15 | 0.946 |
| Hippocampal volume | MCP-1 | -0.016 | -0.052 | 0.020 | 0.386 | 14.787 | 0.393 | 8 | 0.906 |
| Alzheimer's disease | TRAIL | 0.029 | -0.010 | 0.068 | 0.143 | 23.521 | 0.657 | 28 | 0.906 |
| Hippocampal volume | TRAIL | -0.016 | -0.038 | 0.007 | 0.181 | 13.184 | 0.948 | 24 | 0.906 |
| Alzheimer's disease | PDGF-bb | -0.021 | -0.091 | 0.049 | 0.550 | 13.461 | 0.336 | 13 | 0.906 |
| Hippocampal volume | PDGF-bb | -0.028 | -0.070 | 0.014 | 0.189 | 2.512 | 0.961 | 9 | 0.906 |
| Alzheimer's disease | IP-10 | 0.060 | -0.087 | 0.206 | 0.426 | 2.461 | 0.117 | 2 | 0.906 |
| Hippocampal volume | IP-10 | -0.041 | -0.122 | 0.041 | 0.329 | 0.017 | 0.896 | 8 | 0.906 |
| Alzheimer's disease | IL-18 | 0.025 | -0.027 | 0.077 | 0.353 | 13.792 | 0.245 | 12 | 0.906 |
| Hippocampal volume | IL-18 | 0.010 | -0.038 | 0.059 | 0.672 | 14.975 | 0.036 | 8 | 0.911 |
| Alzheimer's disease | SCGF-b | -0.012 | -0.078 | 0.053 | 0.712 | 7.217 | 0.407 | 8 | 0.937 |
| Hippocampal volume | SCGF-b | 0.033 | -0.025 | 0.091 | 0.265 | 14.800 | 0.039 | 3 | 0.906 |
| Alzheimer's disease | IL-2ra | -0.009 | -0.067 | 0.049 | 0.761 | 5.898 | 0.052 | 3 | 0.946 |
| Hippocampal volume | IL-2ra | 0.013 | -0.022 | 0.048 | 0.483 | 5.175 | 0.075 | 5 | 0.906 |
| Alzheimer's disease | CTACK | 0.019 | -0.044 | 0.081 | 0.553 | 10.021 | 0.075 | 6 | 0.906 |
| Hippocampal volume | CTACK | 0.000 | -0.074 | 0.075 | 0.994 | 13.631 | 0.009 | 2 | 0.996 |
| Alzheimer's disease | IL-17 | -0.018 | -0.297 | 0.262 | 0.902 | - | - | 1 | 0.977 |
| Hippocampal volume | IL-17 | 0.134 | -0.031 | 0.300 | 0.111 | - | - | 1 | 0.906 |
| Alzheimer's disease | IL-13 | 0.021 | -0.037 | 0.080 | 0.478 | 1.420 | 0.841 | 5 | 0.906 |
| Hippocampal volume | IL-13 | 0.009 | -0.027 | 0.045 | 0.620 | 4.670 | 0.198 | 4 | 0.906 |
| Alzheimer's disease | HGF | -0.038 | -0.188 | 0.113 | 0.626 | 0.078 | 0.780 | 2 | 0.906 |
| Hippocampal volume | HGF | 0.087 | -0.007 | 0.181 | 0.070 | 0.047 | 0.828 | 2 | 0.906 |
| Alzheimer's disease | IL-10 | -0.034 | -0.122 | 0.054 | 0.454 | 1.897 | 0.387 | 3 | 0.906 |
| Hippocampal volume | IL-10 | 0.018 | -0.034 | 0.071 | 0.494 | 4.515 | 0.105 | 3 | 0.906 |
| Alzheimer's disease | IL-16 | -0.014 | -0.077 | 0.050 | 0.675 | 0.275 | 0.871 | 3 | 0.911 |
| Hippocampal volume | IL-16 | -0.004 | -0.042 | 0.035 | 0.855 | 5.397 | 0.067 | 3 | 0.975 |
| Alzheimer's disease | IL-7 | -0.054 | -0.141 | 0.033 | 0.226 | 0.304 | 0.581 | 2 | 0.906 |
| Hippocampal volume | IL-7 | 0.003 | -0.051 | 0.056 | 0.926 | - | - | 1 | 0.977 |
| Alzheimer's disease | TNF-β | -0.041 | -0.110 | 0.028 | 0.241 | 1.276 | 0.528 | 3 | 0.906 |
| Hippocampal volume | TNF-β | -0.004 | -0.072 | 0.065 | 0.919 | - | - | 1 | 0.977 |
| Alzheimer's disease | MIF | 0.022 | -0.163 | 0.208 | 0.812 | - | - | 1 | 0.953 |
| Hippocampal volume | MIF | -0.043 | -0.155 | 0.068 | 0.446 | - | - | 2 | 0.906 |
| Alzheimer's disease | BNGF | 0.016 | -0.175 | 0.208 | 0.867 | - | - | 1 | 0.975 |
| Hippocampal volume | BNGF | 0.072 | -0.044 | 0.188 | 0.222 | - | - | 1 | 0.906 |
| Alzheimer's disease | SCF | 0.047 | -0.147 | 0.241 | 0.635 | 2.067 | 0.151 | 2 | 0.906 |
| Hippocampal volume | SCF | 0.135 | 0.013 | 0.257 | 0.031 | 3.456 | 0.063 | 1 | 0.552 |
| Alzheimer's disease | IL-5 | -0.105 | -0.294 | 0.084 | 0.278 | - | - | 1 | 0.906 |
| Hippocampal volume | IL-5 | 0.004 | -0.106 | 0.114 | 0.941 | - | - | 1 | 0.977 |
| Alzheimer's disease | IL-1 | 0.085 | -0.140 | 0.310 | 0.461 | - | - | 1 | 0.906 |
| Hippocampal volume | IL-1 | -0.037 | -0.173 | 0.099 | 0.596 | - | - | 1 | 0.906 |
| Alzheimer's disease | ICAM-1 | 0.002 | -0.845 | 0.850 | 0.996 | 5.174 | 0.160 | 4 | 0.996 |
| Hippocampal volume | ICAM-1 | 1.066 | 0.200 | 1.932 | 0.016 | 2.2641 | 0.322 | 3 | 0.552 |

Shown are the results derived from the secondary inverse variance-weighted meta-analysis. Estimate denotes log odds ratio for Alzheimer’s disease and mean difference for hippocampal volume. MIG indicates monokine induced by gamma interferon indicates; MIP-1b, macrophage inflammatory protein 1 beta; VEGF, vascular endothelial growth factor; IL, interleukin; IL, interleukin; GRO-α, growth-regulated oncogene alpha; MCP-1, monocyte chemoattractant protein-1; TRAIL, TNF-related apoptosis-inducing ligand; PDGF-bb, platelet-derived growth factor-bb; IP-10, interferon gamma-induced protein 10; SCGF-b, stem cell growth factor beta; CTACK, cutaneous T-cellattracting chemokine; HGF, hepatocyte growth factor; TNF-related apoptosis-inducing ligand; MIF, macrophage migration inhibitory factor; BNGF indicates beta nerve growth factor; SCF, stem cell factor; ICAM-1, Intercellular Adhesion Molecule 1.

| **Table S3. Alternative tests for markers showing either significant or suggestive associations or significant heterogeneity in the primary IVW MR analysis.** | | | | | | | | | | | |
| --- | --- | --- | --- | --- | --- | --- | --- | --- | --- | --- | --- |
| **Exposure** | **Outcome** | **MR test** | **Estimate*** | **95% CI** | | ***P*-value** | **Heterogeneity** | | **Cochran *Q*-derived *P*** | **SNPs** | **FDR-corrected *P*** |
| Platelet count | Alzheimer’s disease | Random-effect IVW | 0.005 | -0.068 | 0.077 | 0.895 | 24 | | 0.000 | 158 | 1.00 |
|  |  | Weighted median | -0.043 | -0.134 | 0.048 | 0.358 |  | |  |  |  |
|  |  | Weighted mode | -0.062 | -0.174 | 0.050 | 0.279 |  | |  |  |  |
|  |  | MR-Egger | -0.126 | -0.289 | 0.037 | 0.131 |  | |  |  |  |
|  |  | ConMix | -0.060 | -0.110 | 0.000 | 0.083 |  | |  |  |  |
|  |  | MR-PRESSO raw | 0.014 | -0.063 | 0.092 | 0.720 |  | |  |  |  |
|  |  | MR-PRESSO corrected | -0.011 | -0.078 | 0.055 | 0.737 |  | |  |  |  |
|  | Hippocampal volume | Random-effect IVW | -0.018 | -0.059 | 0.023 | 0.393 | 204 | | 0.002 | 151 | 0.84 |
|  |  | Weighted median | -0.016 | -0.077 | 0.044 | 0.594 |  | |  |  |  |
|  |  | Weighted mode | -0.035 | -0.114 | 0.044 | 0.390 |  | |  |  |  |
|  |  | MR-Egger | -0.013 | -0.102 | 0.076 | 0.778 |  | |  |  |  |
|  |  | ConMix | -0.010 | -0.050 | 0.020 | 0.513 |  | |  |  |  |
|  |  | MR-PRESSO raw | -0.019 | -0.061 | 0.024 | 0.385 |  | |  |  |  |
|  |  | MR-PRESSO corrected | -0.013 | -0.054 | 0.029 | 0.549 |  | |  |  |  |
| Eosinophil count | Hippocampal volume | Random-effect IVW | 0.016 | -0.041 | 0.073 | 0.579 | 117 | | 0.034 | 92 | 0.86 |
|  |  | Weighted median | 0.018 | -0.062 | 0.099 | 0.659 |  | |  |  |  |
|  |  | Weighted mode | -0.063 | -0.277 | 0.150 | 0.563 |  | |  |  |  |
|  |  | MR-Egger | 0.024 | -0.113 | 0.162 | 0.730 |  | |  |  |  |
|  |  | ConMix | 0.040 | -0.040 | 0.090 | 0.633 |  | |  |  |  |
|  |  | MR-PRESSO raw | 0.015 | -0.043 | 0.073 | 0.613 |  | |  |  |  |
|  |  | MR-PRESSO corrected | 0.033 | -0.021 | 0.087 | 0.231 |  | |  |  |  |
| Basophil count | Alzheimer’s disease | Random-effect IVW | 0.158 | -0.066 | 0.383 | 0.167 | 6 | | 0.013 | 36 | 0.71 |
|  |  | Weighted median | 0.226 | -0.067 | 0.519 | 0.131 |  | |  |  |  |
|  |  | Weighted mode | 0.527 | -0.046 | 1.100 | 0.080 |  | |  |  |  |
|  |  | MR-Egger | 0.265 | -0.357 | 0.887 | 0.404 |  | |  |  |  |
|  |  | ConMix | 0.300 | 0.030 | 0.560 | 0.028 |  | |  |  |  |
|  |  | MR-PRESSO | No significant outliers | |  |  |  | |  |  |  |
| Monocyte count | Alzheimer’s disease | Random-effect IVW | -0.033 | -0.112 | 0.045 | 0.406 | 15 | | 0.008 | 116 | 0.84 |
|  |  | Weighted median | -0.043 | -0.155 | 0.07 | 0.457 |  | |  |  |  |
|  |  | Weighted mode | -0.034 | -0.176 | 0.109 | 0.646 |  | |  |  |  |
|  |  | MR-Egger | 0.012 | -0.141 | 0.165 | 0.878 |  | |  |  |  |
|  |  | ConMix | -0.040 | -0.120 | 0.040 | 0.340 |  | |  |  |  |
|  |  | MR-PRESSO raw | -0.034 | -0.114 | 0.046 | 0.406 |  | |  |  |  |
|  |  | MR-PRESSO corrected | -0.051 | -0.128 | 0.026 | 0.198 |  | |  |  |  |
|  | Hippocampal volume | Random-effect IVW | 0.011 | -0.039 | 0.060 | 0.671 | 125 | | 0.047 | 101 | 0.93 |
|  |  | Weighted median | 0.044 | -0.019 | 0.106 | 0.169 |  | |  |  |  |
|  |  | Weighted mode | 0.032 | -0.053 | 0.117 | 0.463 |  | |  |  |  |
|  |  | MR-Egger | 0.016 | -0.095 | 0.127 | 0.780 |  | |  |  |  |
|  |  | ConMix | 0.010 | -0.030 | 0.050 | 0.703 |  | |  |  |  |
|  |  | MR-PRESSO raw | 0.012 | -0.039 | 0.063 | 0.651 |  | |  |  |  |
|  |  | MR-PRESSO corrected | 0.020 | 0.067 | 0.067 | 0.401 |  | |  |  |  |
| Lymphocyte count | Alzheimer’s disease | Random-effect IVW | 0.034 | -0.070 | 0.138 | 0.521 | 13 | | 0.009 | 94 | 0.84 |
|  |  | Weighted median | 0.039 | -0.119 | 0.197 | 0.629 |  | |  |  |  |
|  |  | Weighted mode | 0.081 | -0.106 | 0.267 | 0.398 |  | |  |  |  |
|  |  | MR-Egger | 0.179 | -0.106 | 0.463 | 0.218 |  | |  |  |  |
|  |  | ConMix | 0.010 | -0.090 | 0.110 | 0.898 |  | |  |  |  |
|  |  | MR-PRESSO | No significant outliers | |  |  |  | |  |  |  |
|  | Hippocampal volume | Random-effect IVW | 0.023 | -0.041 | 0.086 | 0.483 | 117 | | 0.012 | 86 | 0.84 |
|  |  | Weighted median | 0.048 | -0.031 | 0.127 | 0.237 |  | |  |  |  |
|  |  | Weighted mode | 0.058 | -0.060 | 0.175 | 0.340 |  | |  |  |  |
|  |  | MR-Egger | 0.045 | -0.129 | 0.22 | 0.613 |  | |  |  |  |
|  |  | ConMix | 0.060 | 0.000 | 0.120 | 0.061 |  | |  |  |  |
|  |  | MR-PRESSO | No significant outliers | |  |  |  | |  |  |  |
| CD4 to 8 ratio | Alzheimer’s disease | Random-effect IVW | 0.042 | -0.092 | 0.176 | 0.539 | 12 | | 0.006 | 4 | 0.84 |
|  |  | Weighted median | 0.036 | -0.05 | 0.122 | 0.410 |  | |  |  |  |
|  |  | Weighted mode | 0.002 | -0.099 | 0.102 | 0.977 |  | |  |  |  |
|  |  | MR-Egger | 0.027 | -0.426 | 0.48 | 0.906 |  | |  |  |  |
|  |  | ConMix | 0.000 | -0.130 | 0.280 | 1.000 |  | |  |  |  |
|  |  | MR-PRESSO raw | 0.052 | -0.089 | 0.194 | 0.520 |  | |  |  |  |
|  |  | MR-PRESSO corrected | 0.003 | -0.105 | 0.112 | 0.959 |  | |  |  |  |
| Fibrinogen | Alzheimer’s disease | Random-effect IVW | 0.479 | -0.18 | 1.139 | 0.154 | 88 | | 0.001 | 53 | 0.71 |
|  |  | Weighted median | 0.789 | 0.001 | 1.576 | 0.050 |  | |  |  |  |
|  |  | Weighted mode | 0.717 | -0.187 | 1.621 | 0.126 |  | |  |  |  |
|  |  | MR-Egger | 0.452 | -1.230 | 2.134 | 0.598 |  | |  |  |  |
|  |  | ConMix | 0.780 | 0.140 | 1.460 | 0.019 |  | |  |  |  |
|  |  | MR-PRESSO raw | 0.608 | -0.184 | 1.401 | 0.138 |  | |  |  |  |
|  |  | MR-PRESSO corrected | 0.321 | -0.472 | 1.113 | 0.268 |  | |  |  |  |
| CRP | Alzheimer’s disease | Random-effect IVW | 0.101 | 0.010 | 0.191 | 0.029 | 16 | | 0.852 | 24 | 0.42 |
|  |  | Weighted median | 0.070 | -0.050 | 0.189 | 0.253 |  | |  |  |  |
|  |  | Weighted mode | 0.071 | -0.060 | 0.202 | 0.285 |  | |  |  |  |
|  |  | MR-Egger | 0.086 | -0.104 | 0.234 | 0.452 |  | |  |  |  |
|  |  | ConMix | 0.100 | 0.020 | 0.190 | 0.028 |  | |  |  |  |
|  |  | MR-PRESSO | No significant outlier | | | | |  |  |  |  |
| Lp-PLA2 activity | Hippocampal volume | Random-effect IVW | -0.316 | -0.715 | 0.084 | 0.121 | 19 | | 0.004 | 7 | 0.71 |
|  |  | Weighted median | -0.285 | -0.620 | 0.049 | 0.094 |  | |  |  |  |
|  |  | Weighted mode | -0.133 | -0.668 | 0.401 | 0.642 |  | |  |  |  |
|  |  | MR-Egger | -0.882 | -2.194 | 0.430 | 0.188 |  | |  |  |  |
|  |  | ConMix | -0.180 | -0.440 | 0.170 | 0.427 |  | |  |  |  |
|  |  | MR-PRESSO raw | -0.343 | -0.751 | 0.066 | 0.151 |  | |  |  |  |
|  |  | MR-PRESSO corrected | -0.089 | -0.410 | 0.233 | 0.612 |  | |  |  |  |

Methods used to test for associations and for violations of the Mendelian randomization assumptions. IVW, inverse variance–weighted; MR, Mendelian randomization; ConMix, contamination mixture method; MR-PRESSO, Mendelian Randomization Pleiotropy Residual Sum and Outlier; SNP, single-nucleotide polymorphism; FDR, false-discovery rate, CRP, C-reactive protein; Lp-PLA2, Lipoprotein-associated phospholipase A2. *Log odds ratio for Alzheimer’s disease and mean difference for hippocampal volume.
